# Perspectives and design considerations of capillary-driven artificial trees for fast dewatering processes

**DOI:** 10.1038/s41598-021-88006-z

**Published:** 2021-04-21

**Authors:** Jongho Lee

**Affiliations:** Department of Civil Engineering, University of British Columbia, Vancouver, BC V6T 1Z4 Canada

**Keywords:** Chemical engineering, Civil engineering, Mechanical engineering

## Abstract

Recent progresses on nanocapillary-driven water transport under metastable conditions have substantiated the potential of artificial trees for dewatering applications in a wide pressure range. This paper presents a comprehensive performance analysis of artificial trees encompassing the principle for negative capillary pressure generation; impacts of structural, compositional, and environmental conditions on dewatering performance; and design considerations. It begins by delineating functionalities of artificial trees for evaporation (leaves), conduction (xylem), and filtration (root) of water, in the analogy to natural trees. The analysis revealed that the magnitude of (negative) capillary pressure in the artificial leaves and xylem must be sufficiently large to overcome the osmotic pressure of feed at the root. The required magnitude can be reduced by increasing the osmotic pressure in the artificial xylem conduits, which reduces the risk of cavitation and subsequent blockage of water transport. However, a severe concentration polarization that can occur in long xylem conduits would negate such compensation effect of xylem osmotic pressure, leading to vapor pressure depression at the artificial leaves and therefore reduced dewatering rates. Enhanced Taylor dispersions by increasing xylem conduit diameters are found to alleviate the concentration polarization, allowing for water flux enhancement directly by increasing leaf-to-root membrane area ratio.

## Introduction

As one of the core processes for liquid–solid separation, dewatering is implemented in an ample range of industrial sector, including construction^1,2^, food processing^3,4^, metal recovery^5^, biofuel production^6,7^, and wastewater treatment^8,9^. In light of the increasing practice of zero-liquid discharge, dewatering is also becoming a critical process for addressing environmental concerns arising from industrial wastewater disposal. For instance, the tailing ponds in Alberta, Canada, which contain more than 1.2 trillion litre of the oil–sand produced water, containing toxic chemicals and encompassing over 220 km^2^, are an immense liability to Canadian industries^10,11^. For residential and commercial sectors, effective water removal is integral to stormwater management, as urban flooding may incur sewage overflow, pollutant spread, and outbreak of waterborne diseases^12,13^. While the primary emphasis of dewatering is on the production of concentrated solids or removal of excessive unwanted water, in a broader aspect reusable water can also be produced by dewatering processes when the removed water is captured^14^.

Trees are an effective natural dewatering instrument; up to 97% of absorbed water at roots is transpired to the atmosphere^15^, contributing almost 40% of the annual precipitation over the land^16^. Trees generate a negative capillary pressure in their leaf cells, transduce it through xylem to root cells for water uptake at the root, and conduct water to the leaves, from which water evaporates (i.e., transpiration)^17,18^. The cohesion-tension theory is well-accepted to explain the generating mechanism of negative capillary pressures^17–19^. An air–liquid water interface (meniscus) is formed in channels within the cell walls of leaf mesophyll cells, equivalent to nanopores with diameters of *O*(10 nm)^19,20^. With the meniscus pinned at the periphery of the nanopore, the removed water molecules by transpiration are immediately displaced by water molecules underneath and the chain of water molecules moves upward. Accordingly, a tension force, equivalent to negative pressure, arises in the water molecular chain. The surface tension of water in a nanopore with the diameter of 20 nm can generate a capillary pressure of < − 100 bar, which serves as the driving force for water transport even in giant sequoia (> 100 m in height)^21^.

As salt-tolerant trees, mangroves further extend the use of the negative capillary pressure to the tight control of water and ion uptake, rendering their xylem to be nearly salt-free^22,23^. The blockage of their non-selective route for water and salt passage (apoplastic route) effectively diverts the flow to the selective route (symplastic route); cell lipid membranes and salt-excluding water channels called aquaporins enable a selective water permeation through the root cells. The sufficiently negative capillary pressure generated in mangrove trees overcomes the osmotic pressure in the soil (e.g., ~ 25 bar for seawater), allowing for water uptake in their saline habitat.

This nanocapillary-driven mechanism in trees has been explored for engineering purposes, including energy harvesting^24,25^ and chemical sensing^26,27^. The major challenge lies in that liquid water under negative pressure is at a metastable condition, prone to bubble formation and cavitation (called embolism), which potentially blocks the water passage^28,29^. The susceptibility to embolism has limited experimental demonstrations in artificial platforms to a rather low-pressure range (< ~ 25 bar, seawater osmotic pressure)^24–27,30^. In light of this challenge, Stroock and colleagues demonstrated water transport in microfluidic platforms, driven by negative capillary pressures equivalently down to ~ − 1000 bar^31,32^. In a recent study by the present author and colleagues^33^, an artificial mangrove was presented for hypersaline brine desalination through generating a negative pressure approaching − 300 bar in nanocapillaries and hydrogel, used as artificial leaves. The highly hydrophilic porous silica employed as stem greatly enhanced the stability of water transport under negative pressure, and enabled almost 50 h-long desalination of 5 M NaCl brine, far exceeding the treatable salinity limit (~ 1.3 M NaCl) of conventional reverse osmosis (RO) processes^34^. These recent progresses support the feasibility of the tree-mimicking dewatering in a wide pressure range. Futuristic applications of this dewatering mechanism by constructing artificial trees have also been proposed for large-scale solar desalination^35^ as well as urban stormwater management and surface cooling^33^.

The water flow in nanocapillary-driven artificial trees is a coupled outcome of multiple mass transfer phenomena and multi-phase thermodynamics, including water permeation and solute rejection via steric hindrance or solution-diffusion mechanism (root), convective and diffusive flows (stem and external environment), and liquid–vapor phase equilibrium (leaf). To author’s knowledge, previous studies are primarily focused on local transfer phenomena, which calls for a systematic analysis that accounts for interconnected, multiple transfer phenomena and provides guidelines in designing artificial trees for engineering systems. This paper conducts a systematic modelling on the water transport in an artificial tree of any arbitrary scale through coupling the transfer phenomena in dense/porous media and two-phase thermodynamics, and aims to present the potential of the tree-mimicking, passive and fast dewatering process for large-scale engineering and environmental applications. The paper begins with delineating the functional similarities of water transport between a natural (mangrove) tree and the artificial tree, followed by analyzing the impacts of structural, compositional, and environmental conditions on the dewatering performance. Finally, perspectives and key design considerations are discussed.

## Modelling water transport in artificial trees

**Figure 1:**
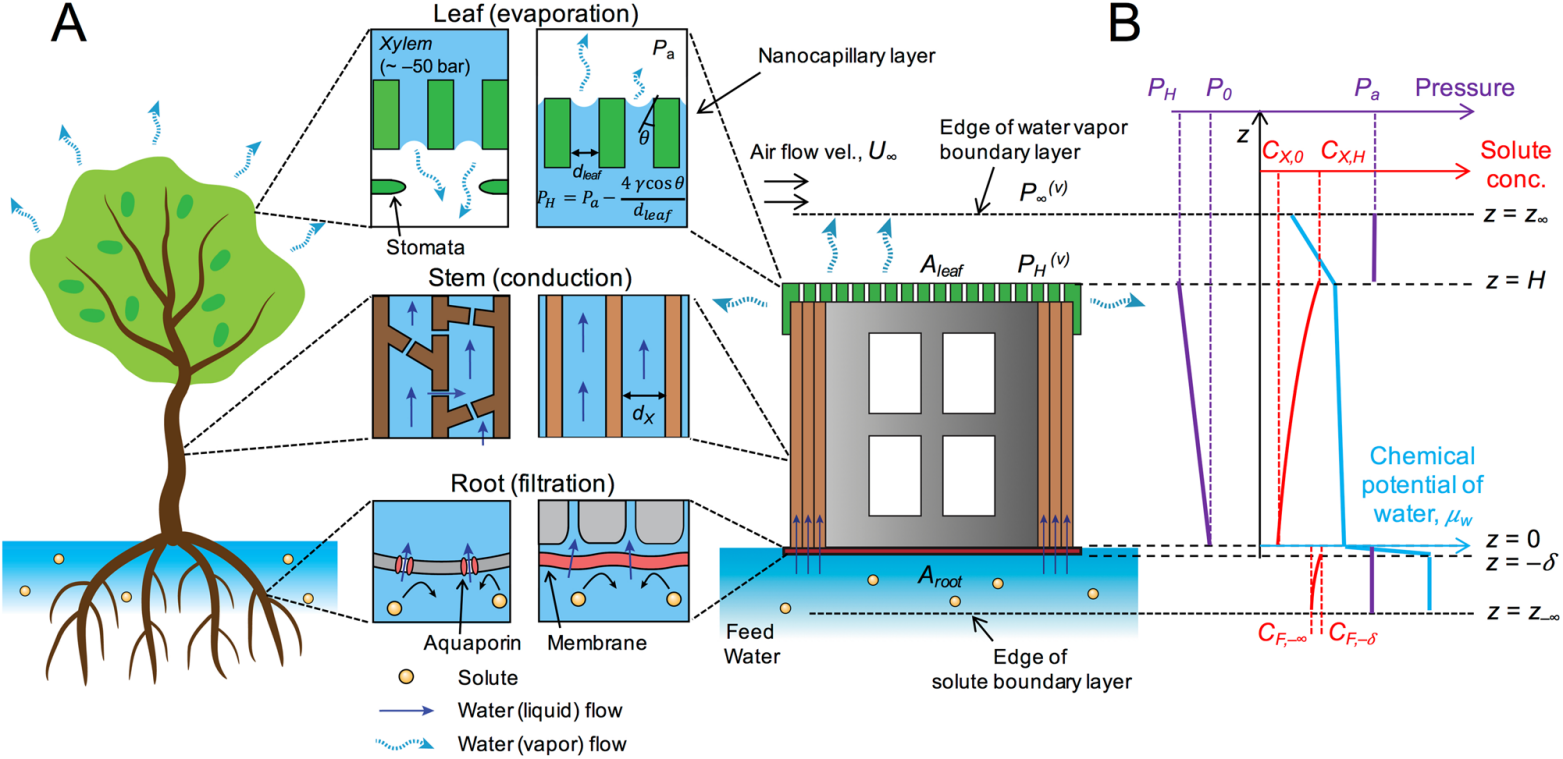
Overview of dewatering process by an artificial tree. (**A**) Schematic illustrations of a natural mangrove tree and an artificial tree for dewatering process, and their similarities in functions: filtration (root), conduction (stem), and evaporation (leaf) of water. (**B**) Distributions of chemical potential of water, pressure, and solute concentration through the artificial tree at different locations.

A dewatering process by natural (mangrove) trees may be simplified into three steps: water filtration by root, conduction through the stem, and transpiration at the leaves (Fig. 1A). As an illustrative example of nanocapillary-driven large-scale dewatering processes^33,35^, we take an artificial tree hypothetically constructed on a building, potentially for urban stormwater management, consisting of the solute rejecting membrane at the foundation, hydrophilic porous conduit, and a nanoporous layer at the roof and the wall surface. Analyses for other environmental applications such as dewatering of high salinity brine or tailing ponds can readily be derived from this illustration. A porous material in the building wall (xylem in the stem) conducts water to the evaporative surfaces that comprise nanopores on the building walls and roofs (leaves). For simplicity, several assumptions are made: (1) xylem conduits are bundles of cylindrical tubes with a uniform diameter; (2) the total cross-section area of xylem conduits is equated to the root membrane area; (3) water transport through all the artificial tree components is assumed to occur under isothermal condition; (4) any solutes in the artificial tree and in the water on the ground (feed) are modelled as NaCl due to its prevalence in natural environments and well-documented osmotic pressure data; and (5) a complete solute rejection at the root membrane is assumed.

Macroscopically, the gradient of chemical potential ($\mu$) through the artificial tree is the driving force for water transport from the feed to the ambient (Fig. 1B). Microscopically, a capillary pressure generated in the leaf pores exerts a mechanical force to drive the water through the root membrane and xylem conduits in the stem. The pressure discontinuity across the liquid–vapor interface (meniscus), formed inside the leaf pore, results in a negative, capillary pressure described by the Young–Laplace equation^19,33^:

1 $$P_{H} - P_{a} = - \frac{4\gamma \cos \theta }{{d_{leaf} }}$$

where *P*_H_ and *P*_a_ are the hydraulic pressure in the leaf pore (at $z = H$) and the ambient pressure, respectively; $\gamma$ and $\theta$ are the surface tension of water and the contact angle between the meniscus and the pore wall surface, respectively. The contact angle ($\theta$) serves as ‘knob’ of the magnitude of $P_{H}$, and the largest pressure discontinuity occurs when $\theta$ becomes the receding contact angle ($\theta_{min}$)^19,33^. For highly hydrophilic pore materials, $\theta_{min} = 10^\circ$ is assumed.

For mild evaporative fluxes from leaf pores, a near-equilibrium of liquid and vapor phases is satisfied. Taking water vapor as an ideal gas, the chemical potentials of water in vapor ($\mu_{H}^{\left( v \right)}$) and liquid ($\mu_{H}^{\left( l \right)}$) phases are expressed as follows^36–38^ (Supplementary note S1):

2 $$\mu_{H}^{\left( v \right)} = \mu^{0} \left( T \right) + RT\ln \frac{{P_{H}^{\left( v \right)} }}{{P_{sat}^{0} \left( T \right)}}$$

3 $$\mu_{H}^{\left( l \right)} = \mu^{0} \left( T \right) - {\Pi }_{H} \overline{v}_{w} + \left[ {P_{H} - P_{sat}^{0} \left( T \right)} \right]\overline{v}_{w} + \rho_{w} gH\overline{v}_{w}$$

Here *R*, *T*, and *g* are the universal gas constant, temperature, and gravitational acceleration, respectively; $\rho_{w}$ and $\overline{v}_{w}$ are the density and the molar volume of liquid water; $\mu^{0} \left( T \right)$ is the chemical potential of water at the reference state (i.e., pure water with a flat liquid–vapor interface and under its saturation vapor pressure, $P_{sat}^{0} (T)$). The second term in the right-hand-side of Eq. (3) stands for the contribution to the chemical potential by the osmotic pressure (${\Pi }_{H}$) of the liquid phase in the xylem, the third term by hydraulic pressure (*P*_H_), and the last term by gravity.

The evaporative mass flux of water vapor from the leaf pores, *J*_w_^(v)^, is expressed as follows:

4 $$J_{w}^{\left( v \right)} = k_{leaf} \left( {P_{H}^{\left( v \right)} - P_{\infty }^{\left( v \right)} } \right)$$

where *P*_H_^(v)^ and *P*_∞_^(v)^ are the saturation vapor pressure at the meniscus ($z = H$) and the partial pressure of vapor in the far ambient ($z = z_{\infty }$), respectively; $k_{leaf}$ is the mass transfer coefficient. For a given root membrane area (*A*_root_), the mass flux of liquid water through the root membrane (*J*_w_) can be amplified by increasing the leaf-to-root area ratio ($A_{R} \equiv A_{leaf} /A_{root} )$: $J_{w} = A_{R} J_{w}^{\left( v \right)}$.

Requiring $\mu_{H}^{\left( v \right)} = \mu_{H}^{\left( l \right)}$ and combining with Eq. (4), we can connect the hydraulic pressure in the leaf pore (*P*_H_), which is negative in value, with external conditions of the environment ($P_{\infty }^{\left( v \right)}$, *T*, and *k*_leaf_), the structural conditions (*H* and *A*_R_), and the solution composition in the xylem (${\Pi }_{H}$):

5 $$P_{H} = P_{sat}^{0} \left( T \right) + \frac{RT}{{\overline{v}_{w} }}\ln \frac{{P_{\infty }^{\left( v \right)} + J_{w} /\left( {A_{R} k_{leaf} } \right)}}{{P_{sat}^{0} \left( T \right)}} + {\Pi }_{H} - \rho_{w} gH$$

The hydraulic pressure at the interface of the root membrane and the stem, $P_{0}$, can then be obtained through the momentum balance:

6 $$P_{0} = P_{H} + \left( {\frac{{32\eta J_{w} }}{{\rho_{w} d_{X}^{2} }} + \rho_{w} g} \right)H$$

where $\eta$ is the dynamic viscosity of liquid water and *d*_X_ is the diameter of xylem conduit. The first and the second terms in the parenthesis stand for pressure drop by viscous loss and hydrostatic pressure, respectively.

The water flux across the salt-rejecting root membrane, such as RO membrane, can be estimated using the solution-diffusion model^39^:

7 $$J_{w} = A_{m} \left( {\Delta P - \Delta {\Pi }} \right)$$

Here *A*_m_ is the water permeability; $\Delta P = P_{a} - P_{0}$ and $\Delta {\Pi } = {\Pi }_{ - \delta } - {\Pi }_{0}$; ${\Pi }_{ - \delta }$ and ${\Pi }_{0}$ are the osmotic pressure at the feed-root membrane interface and the xylem-root membrane interface, respectively. It is apparent that *J*_w_ becomes positive (i.e., dewatering) only when $\Delta P > \Delta {\Pi }$. When *J*_w_ > 0, the solute concentration (${\text{C}}_{F, - \delta }$) and the osmotic pressure (${\Pi }_{ - \delta }$) at the feed-root membrane interface is increased from those in the bulk feed (${\text{C}}_{F, - \infty }$ and ${\Pi }_{ - \infty }$) due to the concentration polarization in the feed (Supplementary note S1).

When solutes are present in the xylem conduit, the solute concentrations at the xylem interface with the root membrane (*C*_X,0_) and with the leaf pore (*C*_X,H_) can be determined by the balance between the convective and diffusive salt fluxes inside the xylem conduit (Supplementary note S1 and S3):

8 $$C_{X,0} = \overline{C}_{X} \frac{{J_{w} H}}{{\rho_{w} D_{eff} }}\left[ {exp\left( {\frac{{J_{w} H}}{{\rho_{w} D_{eff} }}} \right) - 1} \right]^{ - 1}$$

9 $$C_{X,H} = C_{X,0} exp\left( {\frac{{J_{w} H}}{{\rho_{w} D_{eff} }}} \right)$$

where $\overline{C}_{X}$ is a volume-averaged concentration in the xylem; *D*_eff_ stands for an effective diffusion coefficient that accounts for both molecular diffusion and Taylor dispersion. The osmotic pressures in the xylem, ${\Pi }_{0}$ (at $z = 0$) and ${\Pi }_{H}$ (at $z = H$), were calculated using a commercial software (OLI Systems, Morris Plains, NJ) as the function of the solute (NaCl) concentrations $C_{X,0}$ and $C_{X,H}$. These coupled Eqs. (2)–(9) are solved simultaneously to find *J*_w_ by the Newton–Raphson iterative method^40^ (Supplementary note S1). The nomenclature of all variables used is summarized in Supplementary note Table S1.

## Water evaporation from leaves

### Dependency of water evaporation rate on external conditions of the environment

At the artificial leaves, the higher partial pressure of vapor at the meniscus in the leaf pore (at $z = H$) than that in the far ambient (at $z = z_{\infty }$), which is determined by the relative humidity (*RH*) of the ambient, drives the transport of water molecules across a boundary layer of water vapor concentration. Under the turbulent flow condition, the following Sherwood correlation may be used to obtain $k_{leaf}$^41^:

10 $$Sh_{leaf} = 0.037Sc^{1/3} Re_{L}^{4/5}$$

Here $Sh_{leaf} \equiv k_{leaf} \left( {\frac{RT}{{\varepsilon M_{w} }}} \right)\frac{L}{{D_{w}^{\left( v \right)} }}$^33^; *M*_w_ is the molar mass of water; *L* is the characteristic length over which the boundary layer of water vapor concentration develops and $D_{w}^{\left( v \right)}$ is the diffusion coefficient of water vapor; $Sc = \nu_{a} /D_{w}^{\left( v \right)}$ and $Re_{L} = U_{\infty } L/\nu_{a}$, where $\nu_{a}$ is the kinematic viscosity of air. Hereafter, leaf surface porosity (ε) of 0.4, air velocity ($U_{\infty }$) of 1.4 m s^−1^ (= 5 km h^−1^), and feed flow velocity ($U_{ - \infty }$) of 1 m s^−1^ are set for calculation. *L* = 50 m is assumed as a commonly observable length scale of urban buildings.

**Figure 2:**
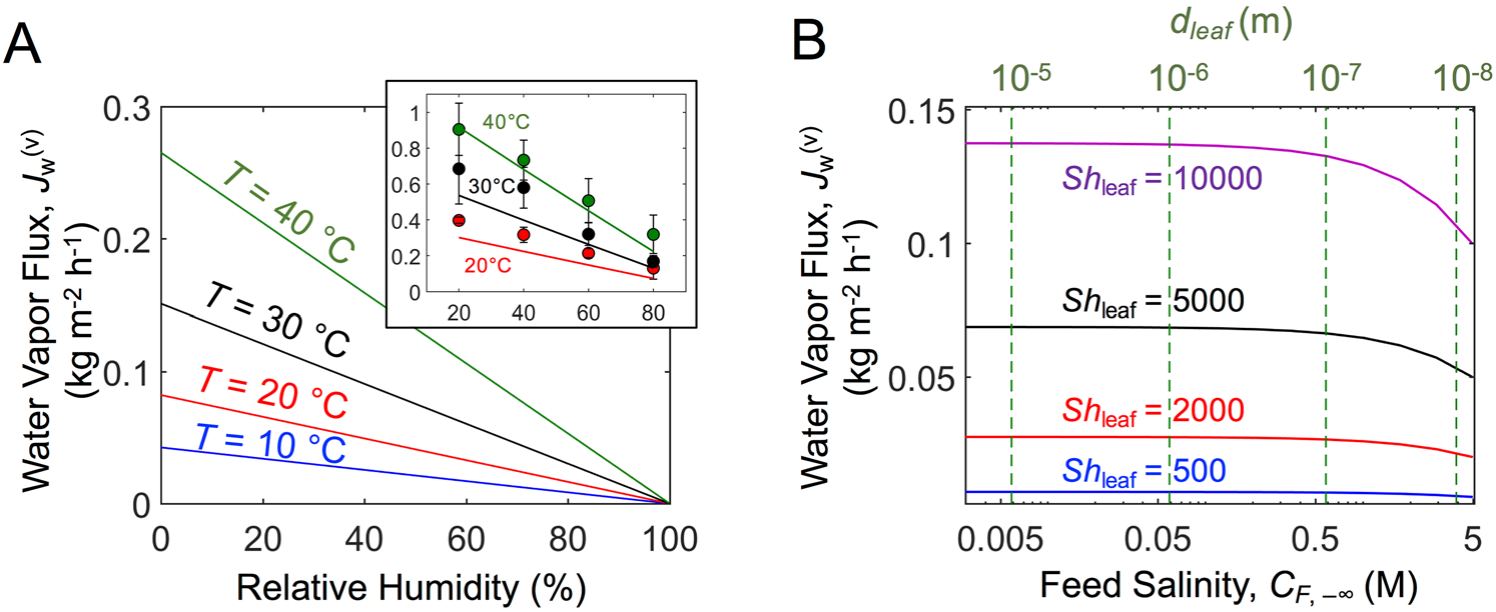
Evaporated water flux from leaves. (**A**) Evaporative water flux at leaves for different relative humidities and temperatures of the environment. Air velocity (*U*_∞_) of 1.4 m s^−1^ (= 5 km h^−1^) is considered. (Inset) Experimental measurement (symbols) and model prediction (Eq. (4), solid lines) of water vapor fluxes for the previously reported lab-scale artificial tree^33^. (**B**) Evaporative water flux for different solute concentrations in the feed water on the ground (at root) and mass transfer coefficients. Here, NaCl is assumed as feed solute, and relative humidity (*RH*) of 0.4 and *T* = 30 °C are considered. The dotted lines indicate the maximum treatable feed soulte concentration, which is determined by the corresponding leaf pore diameter, *d*_leaf_ (Eq. 1), marked on the upper *x*-axis. For all calculations, leaf surface porosity (ε) of 0.4, length scale of the leaf surface (*L*) of 50 m, and feed flow velocity (*U*_−∞_) of 1 m s^−1^ are considered. As a reference, *U*_∞_ = 1.4 m s^−1^ and *L* = 50 m correspond to *Sh*_leaf_ = 6600.

The evaporative water flux (*J*_w_^(v)^) is calculated from Eq. (4), with *RH* expressed as: $RH = P_{\infty }^{\left( v \right)} /P_{sat}^{0} \left( T \right)$. Figure 2A shows the dependency of *J*_w_^(v)^ on the temperature and *RH* in the ambient. The same model (Eq. 4) applied for the lab-scale artificial tree reported by our previous study shows strong agreement with the experimental measurements (Fig. 2A inset)^33^. Given a mass transfer coefficient (*k*_leaf_), as expected, a higher *RH* reduces the vapor pressure difference across the boundary layer and therefore *J*_w_^(v)^ linearly decreases. Also, the elevated saturation vapor pressure at a higher temperature results in the larger evaporative flux. The impact of *k*_leaf_ on the evaporative flux is clear in Fig. 2B, showing that *J*_w_^(v)^ is directly proportional to *k*_leaf_, as apparent from Eq. (4).

### Capillary pressure generated in leaf pores limits the treatable solute concentration of feed

The capillary pressure in the leaf must be sufficiently low to withdraw water from the feed by overcoming its osmotic pressure and to transport water through the stem by overcoming the viscous loss and hydrostatic pressure. The magnitude of capillary pressure increases as the pore size and the contact angle decrease (Eq. 1). Figure 2B also shows the achievable *J*_w_^(v)^ for different feed solute concentrations. The maximum leaf pore diameters (*d*_leaf_) that allow for dewatering of the corresponding feed concentrations are also shown. For example, *d*_leaf_ < 10 nm would be required to overcome the osmotic pressure of 5 M NaCl feed.

It is worth noting that *J*_w_^(v)^ decreases with the feed concentration. The concave curvature of the meniscus in the leaf pore is associated with the depression of saturation vapor pressure on the leaf surface, *P*_H_^(v)^^42,43^, (Supplementary note S1), leading to decrease of *J*_w_^(v)^ (Eq. 4). This water vapor flux reduction due to the vapor pressure depression is more prominent for a larger *Sh*_leaf_ (i.e., larger *k*_leaf_). Given a feed solute concentration, the leaf pore diameter must be small enough to enable dewatering, but the vapor pressure depression reduces the water flux.

## Water conduction through stem

### Solute presence in the artificial xylem reduces the required magnitude of negative pressure and increases the maximum treatable solute concentration of feed

Liquid water at pressure below its saturation vapor pressure is in a metastable condition, and is prone to embolism^28,29^. Hence, artificial xylems are desired to be situated under a less negative pressure^35^. Rearranging Eq. (7) to: $J_{w} = A_{m} \left[ {\left( {P_{a} - {\Pi }_{ - \delta } } \right) - \left( {P_{0} - {\Pi }_{0} } \right)} \right]$, it can be seen that increasing the osmotic pressure in the xylem (${\Pi }_{0}$) can allow for a higher *P*_0_ (i.e., less negative).

**Figure 3:**
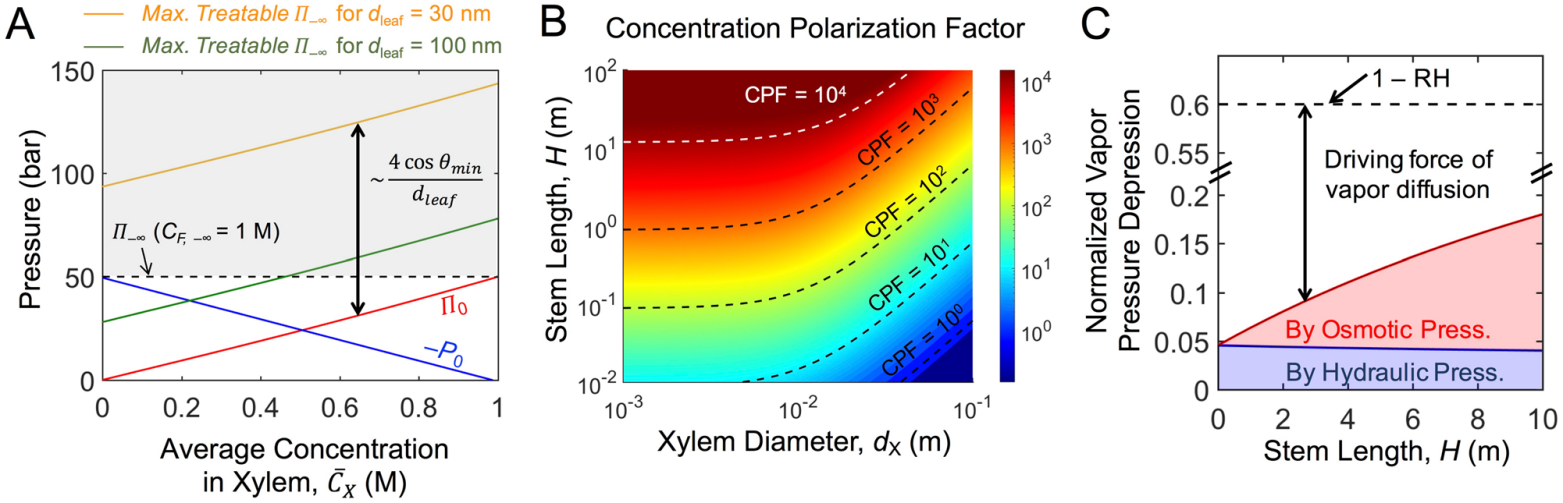
Effects of stem characteristics on water flow through artificial xylems. (**A**) Impact of solute concentration in the xylem on negative hydraulic pressure (− *P*_0_) and osmotic pressure (Π_0_) at the xylem-root membrane interface, and on maximum treatable osmotic pressures of feed (Π_F,−∞_) for two different leaf pore diameters (*d*_leaf_). Shown as a dotted line, feed solute concentration (*C*_F, −∞_) of 1 M NaCl was used to calculate *P*_0_ and Π_0_. The shaded area indicates a feasible range of treatable feed osmotic pressure, which is, $\approx {\Pi }_{0} + \frac{{4\gamma \cos \theta_{min} }}{{d_{leaf} }}$, where $\theta_{min}$ is the receding contact angle in the leaf pore. For *d*_leaf_ = 100 nm, $\overline{C}_{X}$ > 0.47 M is required to recover water from the feed of 1 M NaCl. (**B**) Degree of concentration polarization in the xylem conduit determined by stem length (*H*) and xylem conduit diameter (*d*_X_). Concentration polarization factor (CPF) is defined as CPF ≡ (*C*_X,H_ − *C*_X,0_)/ $\overline{C}_{X}$. (**C**) Vapor pressure depressions on the leaf pore, due to the negative hydraulic pressure (blue shaded area) and the elevated osmotic pressure (red shaded area) by the concentration polarization in the xylem. The vapor pressure depressions are normalized to *P*_sat_^0^(*T*). The dotted line indicates 1 − *RH*, and the gap between the dotted line and the shaded areas represents the driving force for water vapor diffusion from the leaf surface to the ambient. Here, *d*_X_ = 0.01 m is considered for the maximized concentration polarization, as shown in (**B**). For all calculations in (**B**) and (**C**), *C*_F, −∞_ = 1 M NaCl and $\overline{C}_{X}$ = 0.5 mM are considered. *A*_R_ is set as 100 to induce severe concentration polarization.

Figure 3A shows hydraulic or osmotic pressure values required for dewatering 1 M NaCl as a feed for different mean solute concentrations in xylem ($\overline{C}_{X}$). A small length of stem was considered ($H = 1 \,\upmu {\text{m}}$) to neglect any concentration polarization effect inside the xylem conduit, which is discussed in the next section. With ${\Pi }_{ - \delta } \approx {\Pi }_{ - \infty } \approx 50\,{\text{bar}}$ for 1 M NaCl feed, the magnitude of the negative pressure at the xylem-root membrane interface (*P*_0_) decreases as $\overline{C}_{X}$ (and therefore ${\Pi }_{0}$) increases. The maximum osmotic pressure of feed water that can be dewatered is the Laplace pressure added to ${\Pi }_{0}$ (i.e., ${\Pi }_{0} + \frac{{4\gamma \cos \theta_{min} }}{{d_{leaf} }}$). Therefore, an increased xylem solute concentration ($\overline{C}_{X}$) provides more room to dewater the feed of a higher salinity. For example, when *d*_leaf_ = 100 nm, the capillary pressure is not large enough to overcome the osmotic pressure of 1 M NaCl feed. However, dewatering of the feed becomes possible by having $\overline{C}_{X}$ > 0.47 M NaCl. For *d*_leaf_ = 30 nm, dewatering 1 M NaCl is possible even with pure water in the xylem, as ${\Pi }_{0} + \frac{{4\gamma \cos \theta_{min} }}{{d_{leaf} }} \approx 95 \,{\text{bar}} > {\Pi }_{ - \infty } \approx 50 \,{\text{bar}}$. But the maximum treatable solute concentration of the feed can be much more extended.

### Solute presence in the xylem can result in severe concentration polarization

The upward water flow and the presence of solutes in the xylem inevitably causes concentration polarization, resulting in the highest solute concentration in the leaf pore (*C*_X,H_) and the lowest at the xylem-root membrane interface (*C*_X,0_) (Eqs. 8 and 9, and Fig. 1). This concentration polarization causes two negative effects: (1) reducing the osmotic pressure at the xylem-root membrane interface and therefore discounting its compensation effect on reducing the required magnitude of negative pressure; (2) increasing the solute concentration in the leaf pore, which causes the vapor pressure depression and therefore reduces the water vapor flux.

Here, a concentration polarization factor (CPF) is defined as the maximum solute concentration difference normalized by the mean concentration: $CPF \equiv \frac{{C_{X,H} - C_{X,0} }}{{\overline{C}_{X} }}$. Figure 3B shows CPF values for different xylem conduit diameter (*d*_X_) and the stem length (*H*). The CPF values become very large for high *H*, due to the increased path length for diffusion. On the other hand, a larger *d*_X_ incurs an enhanced Taylor dispersion that facilitates the solute diffusion^44^, leading to reduced CPF values. The reduced concentration polarization is more prominent when the water flux across the xylem is higher (Fig. S1). The effect of Taylor dispersion on enhancing water flux is discussed later in detail.

### Severe concentration polarization results in vapor pressure depression, reducing the water flux

A saturation vapor pressure at the meniscus ($P_{H}^{\left( v \right)}$), higher than the partial vapor pressure in the ambient ($P_{\infty }^{\left( v \right)}$), drives the water vapor diffusion from the leaf surface to the ambient. While $P_{\infty }^{\left( v \right)}$ is set by *RH* in the ambient, $P_{H}^{\left( v \right)}$ decreases as the temperature decreases, the solute concentration in the liquid increases, and the hydraulic pressure of the liquid decreases (Supplementary note S1)^42,43^. When the liquid water in the leaf pore is at a largely negative pressure and exerts a large osmotic pressure due to the concentration polarization, *P*_H_^(v)^ is depressed from *P*_sat_^0^(*T*), resulting in a reduced water flux. For $\overline{C}_{X} = 0.5 \,{\text{mM}}$ and $C_{F, - \infty } = 1M$, Fig. 3C depicts the vapor pressure depression normalized to *P*_sat_^0^(*T*), i.e., $\frac{{P_{sat}^{0} \left( T \right) - P_{H}^{\left( v \right)} }}{{P_{sat}^{0} \left( T \right)}}$. It shows fractions of vapor pressure depression by the hydraulic pressure (blue shaded area) and by the osmotic pressure (red shaded area). With *P*_sat_^0^(*T*) being the maximum value of *P*_H_^(v)^, 1 − *RH* represents the normalized maximum driving force for vapor diffusion, i.e., $\frac{{P_{sat}^{0} \left( T \right) - P_{\infty }^{\left( v \right)} }}{{P_{sat}^{0} \left( T \right)}} = 1 - RH$. Owing to the need to overcome the feed osmotic pressure, the hydraulic pressure in the xylem conduit must be at least lower than − 50 bar for 1 M NaCl feed, which depresses ~ 5% of the vapor pressure at the meniscus. For a larger *H*, the solute concentration in the leaf pore increases due to a more severe concentration polarization, and the increased osmotic pressure at the leaf pore leads to a further vapor pressure depression. For instance, with $H = 10 \,{\text{m}}$ and $RH = 0.4$, the total vapor pressure depression accounts for ~ 30% of the maximum driving force (1 − *RH*), and a greater loss of driving force is expected for a higher *RH*.

## Impact of root membranes on water transport

### A high water permeability of root membrane marginally increases water flux, but significantly reduces the required magnitude of negative pressure

**Figure 4:**
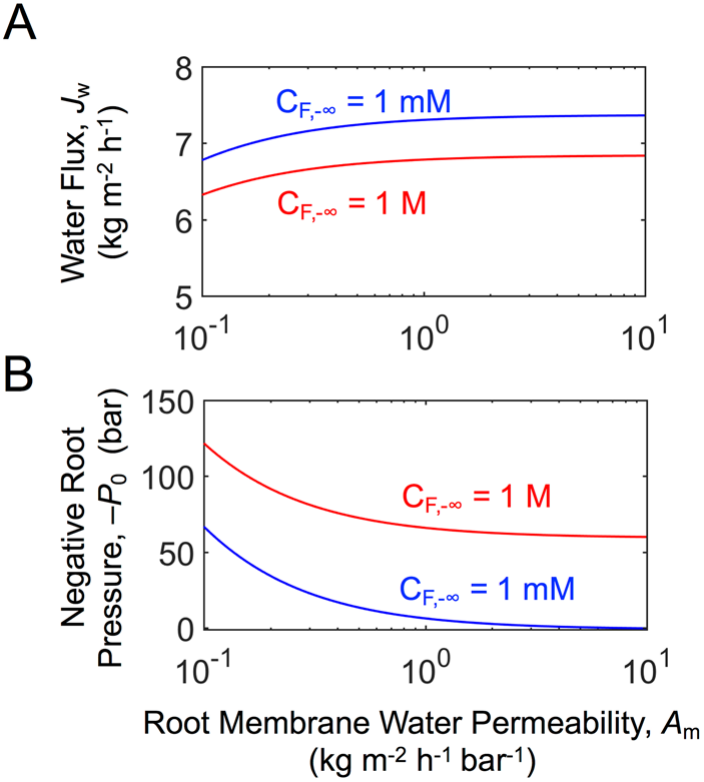
Impact of water permeability of root membrane on water flux (**A**) and on negative pressure at the xylem-root membrane interface **(B)** for two different feed solute concentrations (*C*_F, −∞_) of 1 mM and 1 M NaCl. $\overline{C}_{X}$ = 0.5 mM NaCl is considered such that the maximum concentration of solutes in the xylem does not exceed 5 M. And, *d*_X_ = 0.01 m and *A*_R_ = 100 are considered.

Given a water flux (*J*_w_), a root membrane with lower water permeability poses a greater transport resistance of water, requiring a larger negative pressure at the xylem-root membrane interface (*P*_0_). Figure 4A, B show *J*_w_ and the required *P*_0_, respectively, for different water permeabilities of the root membrane (*A*_m_) and two different feed solute concentrations (*C*_F, −∞_). Leaf-to-root membrane area ratio (*A*_R_) of 100 is considered for a high flux of water withdrawn into the xylem conduit per given external conditions for evaporation. The dependency of water flux on *A*_m_ is rather weak. With *A*_m_ ~ *O*(1 kg m^−2^ h^−1^ bar^−1^ or L m^−2^ h^−1^ bar^−1^) for typical seawater RO membranes^45^, the water flux variation for *A*_m_ in the range of 0.1–10 kg m^−2^ h^−1^ bar^−1^ does not exceed 10%.

On the other hand, the magnitude of *P*_0_ increases by more than 60 bar when *A*_m_ decreases from 1 to 0.1 kg m^−2^ h^−1^ bar^−1^ (Fig. 4B). The requirement of a larger pressure difference across a lower *A*_m_ membrane demands a more negative pressure to be generated by the leaf pores, which in turn increases the cavitation risk in the artificial xylem. Therefore, *A*_m_ values of commercial seawater RO membranes are well-suited for the root membranes without incurring a significant pressure drop.

### A tight membrane is necessary to prevent severe concentration polarization in the xylem conduit

Due to the concentration polarization, it is desirable to prevent solute passage into the xylem, in particular when the stem length is large. In salt-rejecting membranes, a trade-off between water permeability and salt rejection is generally observed^45^. As previously discussed, a very tight (low *A*_m_ and high rejection) membrane likely incurs highly negative pressure in the xylem. Nevertheless, commercial seawater RO membranes, with *A*_m_ of ~ *O*(1 kg m^−2^ h^−1^ bar^−1^), can readily achieve > 99.9% rejection of monovalent salt (such as NaCl). Therefore, when artificial trees target dewatering of urban stormwater, which has typically low solute concentrations, the commonly available seawater RO membranes would suffice. When dewatering of industrial wastewater of high solute concentrations is aimed, higher salt rejecting membranes will be necessary.

## Combined effects of structural properties on water flux

**Figure 5:**
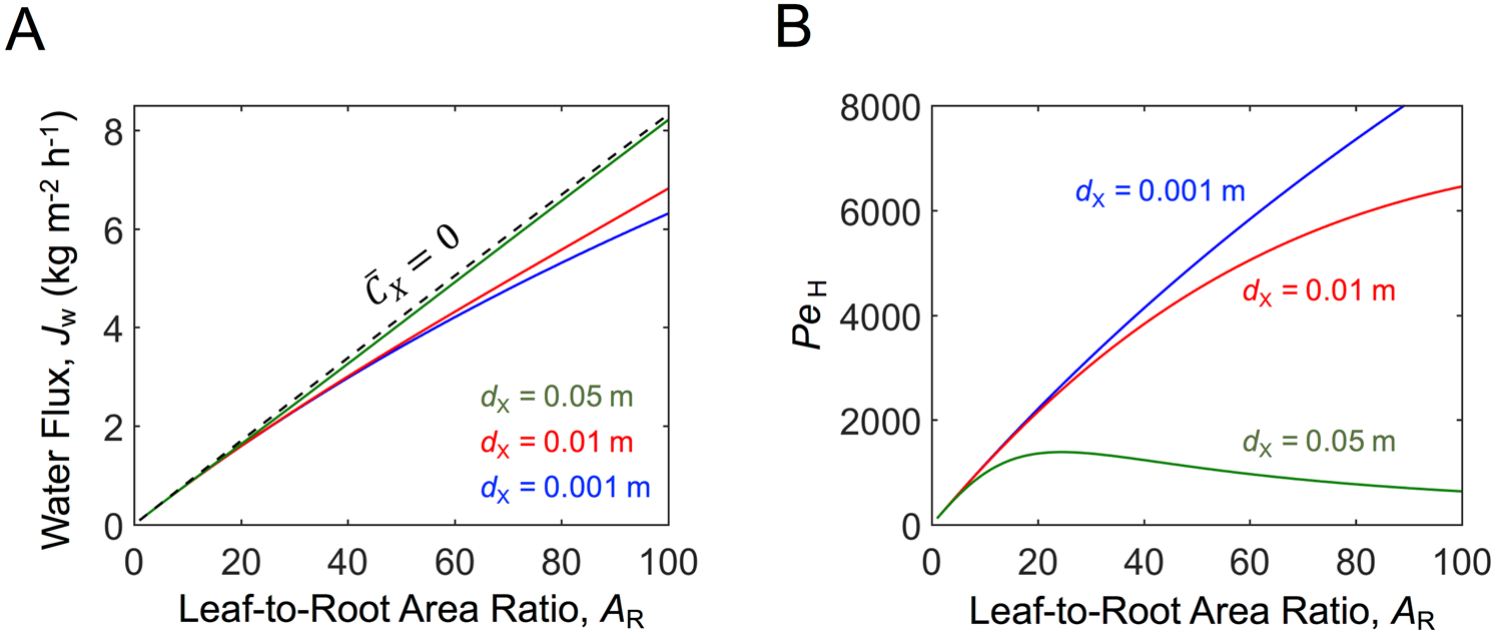
Impact of artificial tree structure on dewatering performance. (**A**) Water flux in the artificial tree for different values of leaf-to-root area ratio (*A*_*R*_ ≡ *A*_leaf_/*A*_root_) and xylem conduit diameter (*d*_X_). The dotted line shows water flux in the absence of solutes in the xylem, where the water flux is independent of value of *d*_X_. (**B**) Dependency of *Péclet* number (*Pe*_H_) on *A*_*R*_ and *d*_X_. A high *Pe*_H_ value implies severe concentration polarization in the xylem. For all calculations in (**A**) and (**B**), *H* = 10 m, *C*_F, −∞_ = 1 M, and $\overline{C}_{X}$ = 0.5 mM are considered.

When external conditions such as temperature, air flow velocity, and *RH* are given, the water flux is mainly determined by combined effects of structural properties of the artificial tree. Fast dewatering requires a large leaf area for evaporation. In fact, the large canopy areas of trees per a given footprint is an integral element for tree-based urban stormwater control^46^. Figure 5A shows the water flux (*J*_w_) dependency on the leaf-to-root membrane area ratio (*A*_R_) for the artificial xylem containing pure water ($\overline{C}_{X} = 0$) and 0.5 mM NaCl, with different xylem conduit diameters (*d*_X_) and the stem length (*H*) of 10 m. As anticipated, for $\overline{C}_{X} = 0$ the water flux linearly increases with *A*_R_. When $\overline{C}_{X} > 0$, however, the degree of water flux increase is reduced owing to the concentration polarization and the resulted vapor pressure depression at the leaf surface. This impact of concentration polarization on the water flux is gradually relieved by increasing the xylem conduit diameter. The degree of concentration polarization can be represented by the Péclet number, *Pe*_H_:

$$Pe_{H} = \frac{{\overline{U}_{X} H}}{{D_{eff} }} \approx \left\{ {\begin{array}{*{20}l} {\frac{{\overline{U}_{X} H}}{{D_{0} }} = Pe_{d} \left( {\frac{H}{{d_{X} }}} \right) \propto \overline{U}_{X} } \hfill & {for\;Pe_{d} \ll 1} \hfill & {(10{\text{a}})} \hfill \\ {\frac{{196\overline{U}_{X} H}}{{D_{0} Pe_{d}^{2} }} = \frac{196}{{Pe_{d} }} \left( {\frac{H}{{d_{X} }}} \right) \propto \overline{U}_{X}^{ - 1} } \hfill & { for\;Pe_{d} \gg 1} \hfill & {(10{\text{b}})} \hfill \\ \end{array} } \right.$$

where $D_{eff} = D_{0} \left( {1 + Pe_{d}^{2} /196} \right)$ and $Pe_{d} = \frac{{\overline{U}_{X} d_{X} }}{{D_{0} }}$^44^. Figure 5B shows the changes in *Pe*_H_ as *A*_R_ increases for different *d*_X_ values. A large *Pe*_H_ implies a convective-dominant flow of solutes, leading to severe concentration polarization. In a low *Pe*_H_ flow, the diffusive flow counterbalances the convective flow, which deters the concentration polarization (Eq. 9) and is ideal to attaining high water fluxes. The impact of *d*_X_ on *Pe*_H_ and therefore on the water flux can be illustrated from two extreme cases. For $Pe_{d} \ll 1$ ($d_{X} < \sim 0.01 {\text{m}}$), *Pe*_H_ linearly increases with the water flow velocity in the xylem ($\overline{U}_{X}$) (Eq. 10a). In this case, a higher $\overline{U}_{X}$ induces a more severe concentration polarization that in turn prevents enhancement of $\overline{U}_{X}$; the enhancement of the water flux by increasing *A*_R_ becomes smaller. At $A_{R} = 100$, for instance, the water flux is ~ 24% less than that from no concentration polarization (dotted line in Fig. 5A), consistent with the reduced driving force by concentration polarization (Fig. 3C).

For $Pe_{d} \gg 1$ ($d_{X} \gg \sim 0.01 {\text{m}}$), on the other hand, *Pe*_H_ is inversely proportional to $\overline{U}_{X}$ (Eq. 10b); an increased flow velocity enhances Taylor dispersion, outweighing the convective flow. Therefore, an increased *A*_R_ will result in a near-proportionally enhanced water flux (Fig. 5A) along with a deterred concentration polarization as represented by the low *Pe*_H_ (Fig. 5B). In these both cases of *Pe*_d_, *Pe*_H_ increases with *H*, inducing a more severe concentration polarization. Nevertheless, for $Pe_{d} \gg 1$, the concentration polarization can be alleviated by employing xylem conduits of a large diameter.

## Design consideration and discussion

Several aspects of driving force, stability, and water flux for attaining effective nanocapillary-driven dewatering are discussed here. First, a sufficiently large negative pressure must be generated to uptake the water from the feed. A lack of driving force cannot lead to water uptake to the leaf pores, and result in drying of the leaf pores and failure of the dewatering process^33^. As the attainable maximum negative pressure is also determined by the receding contact angle (Eq. 1), a high hydrophilicity of the leaf capillary is essential. A suitable leaf capillary layer can be readily constructed using mesoporous hydrophilic materials^47,48^. In case pores are partially dried in such materials, a sizable transport resistance will be added due to the increased path length of vapor transport^49–51^ and will need to be considered for dewatering flow rate estimation. For pores of irregular shape, a geometrical factor should be considered when calculating the capillary pressure^52^. Additionally, it is noted that an increased humidity of the environment by temperature drop in the nocturnal period will likely induce water condensation on the leaf layer in particular when the saturation vapor pressure is depressed, corresponding to a concave curvature of meniscus in the hydrophilic leaf pore. As the excessive water on the leaf surface will evaporate, it would be practically less of a concern, but the water condensation may delay the onset of the dewatering process.

Second, the process stability will critically rely on the suppression of gas bubble formation and subsequent cavitation, as well as on the prevention of bubble expansion through the artificial xylem. As operated under negative pressures, natural trees are constantly at the risk of embolism of xylem conduits^28,29^. Their mechanism to repair xylem embolism is still unclear. However, an onset of cavitation may be deterred in artificial trees through several measures. The inner surface of artificial xylems must exhibit high hydrophilicity, which in turn elevates the free energy barrier for formation of bubble nuclei of critical size prior to an event of cavitation (Supplementary note S2 and Fig. S2)^53^. Likewise, any hydrophobic contaminants must not enter the artificial xylem conduits. A recent study revealed that an entrainment of naturally present lipids significantly increases the chance of cavitation in xylem conduits, due to the preferable bubble nuclei growth on the hydrophobic lipids^54^. For artificial trees, a water-permeable but highly solute-rejecting membrane is essential. Commercial seawater RO membranes effectively reject most of solutes, while exhibiting a sufficient water permeability. Also, the cavitation risk can be reduced by decreasing the necessary magnitude of negative pressure in the xylem. Use of saline solution in the artificial xylem will be an effective strategy for enhancing the stability (or preventing cavitation) when the stem length is small. For a ‘tall’ artificial tree, however, the presence of solutes would induce concentration polarization in the xylem conduit, which potentially negates the compensation effect of elevating the xylem osmotic pressure and results in a decreased water flux owing to the vapor-pressure depression.

In addition, dissolved air in the xylem conduit is another source for xylem embolism in natural trees^52,55^. When the temperature is lowered (e.g., a nocturnal period) and water evaporation is slow, the increased air solubility will likely induce dissolution of air into the artificial xylem through the leaf pores. As recently demonstrated by the author and colleagues, use of a tight hydrogel as the artificial leaf layer may be a suitable strategy to prevent air dissolution while still generating large negative pressures.

It is worth mentioning that natural trees possess a xylem system consisting of segmented conduits, working as a safety device, which ensures water transport even when embolism occurs^56,57^. The conduit segments (tracheids and vessels) are connected to the neighboring segments through finely porous pit membranes^58^. In the event of embolism, the pit membranes allow for local confinement of the bubble in that segment, and prevents the bubble from spreading to the entire xylem network^59,60^. Although in the present study straight cylindrical pores were considered for artificial xylem, water conduits with the small segments delineated with nanoporous membranes, resembling the xylem structure in natural trees, will improve the stability of the dewatering process. Nevertheless, the improved stability will be achieved at the cost of increased hydraulic resistances.

Third, increasing the leaf area for evaporation is a straightforward approach to enable a high water flux (Fig. 5). As shown in the current example of an artificial-tree-transformed-building, a larger leaf surface on the building wall and roof-top surface would be attainable for a taller building with a given footprint on the ground. Even for a 50 story-building (~ 150 m tall), the additional negative pressure required for the water pumping is ~ 15 bar, which can be achieved by employing nanocapillaries with a diameter of ~ *O*(10 nm). A greater leaf area exposed to the air can further be obtained by increasing the surface roughness. To put into perspective, the transpiration rate by natural trees ranges from 10 to 200 kg day^−1^ tree^−1^^46,61^. Assuming 50 m^2^ ground footprint for a single urban tree, an artificial tree occupying the equivalent footprint can enable ~ 500 kg day^−1^ for *A*_R_ of 20 and 6 h of solar irradiation (Fig. 5). An expanded root area by increasing roughness or multiple root ‘branches’ would also enhance the dewatering rate, which would particularly be relevant to the water removal from tailing ponds, concentrating brines for metal recovery, or solar desalination of seawater^35^.

It should be mentioned that although this capillary-driven dewatering is a passive process without requiring any mechanical pumping, the evaporative water flux is essentially limited by the solar energy and heat transferred from the environment. As such, a leaf layer that can effective absorb solar irradiation would further enhance the dewatering rate. For instance, photothermally engineered materials can allow for effective absorption of solar irradiation in a wide wavelength range^62^.

When potentially implemented, this tree-mimicking dewatering requires further considerations of realistic air flows and heat transfer in the environment. In this paper, a single-standing artificial tree was considered for simplicity of analysis. However, when there are multiple artificial trees (for instance, aiming for a Sponge City^33^), the interference of air flows with multiple structures would need to be accounted to estimate the more accurate dewatering rate. Also in other cases such as dewatering of tailing ponds, geographical influence on the air flow would need to be considered. Additionally, the directions and intensity of solar irradiation as well as air flow velocity and humidity have the determining role on the dewatering rate. Hence, for such a large-scale implementation, sophisticated weather models will be necessary to predict the performance.

## Supplementary Information

Supplementary Information.
