# Supplementary material for: Perspectives and design considerations of capillary-driven artificial trees for fast dewatering processes
Source: Sci Rep. 2021 Apr 21;11:8631. doi: 10.1038/s41598-021-88006-z (PMC8060284; doi:10.1038/s41598-021-88006-z)
Supplement: Supplementary file 1 — Supplementary Information. [file 41598_2021_88006_MOESM1_ESM.pdf]

Supplementary Information

Perspectives and Design Considerations of Capillary-Driven Artificial Trees for Fast Dewatering Processes

Scientific Reports

Jongho Lee[1,*]

[1] *Department of Civil Engineering, University of British Columbia, Vancouver, British Columbia, Canada V6T 1Z4*

* Corresponding author:

Jongho Lee, Email: jongho.lee@civil.ubc.ca, Phone: (604) 822-4694

Number of Pages: 15

Number of Figures: 3

Number of Table: 1

Note S1. Modeling details of water transport in artificial trees

1) Leaf pores

The near-equilibrium of water in the liquid and vapor phase at the meniscus (i.e., liquid-vapor interface in the leaf pore) requires equating the chemical potentials of water in the two phases. The chemical potential of water in the vapor phase (at $z = H$) under the ambient pressure (P_a = 1 bar) is given as[1-3]:

$$\mu_H^{(v)} = \mu^0(T) + RT \ln a_w + RT \ln \frac{P_a}{P_{sat}^0(T)} \quad \text{(S1.)}$$

Here, R and T the universal gas constant and temperature, respectively; $\mu^0(T)$ is the chemical potential of water at the reference state, i.e., at saturation vapor pressure ($P_{sat}^0(T)$) and for pure water, and only a function of temperature; the water activity (a_w) is expressed as $a_w = \gamma_w^G n_w^* = \gamma_w^G \frac{P_H^{(v)}}{P_a}$, where γ_w^G is the activity coefficient and n_w^* is the mole fraction of water vapor in air ($n_w^* = \frac{P_H^{(v)}}{P_a}$). $P_H^{(v)}$ is the saturation vapor pressure at the meniscus ($z = H$). At moderate pressure, the water vapor can be assumed an ideal gas, and hence $\gamma_w^G \approx 1$. Then, Eq. (S1) can be rearranged to:

$$\mu_H^{(v)} = \mu^0(T) + RT \ln \frac{P_H^{(v)}}{P_{sat}^0(T)} \quad \text{(S2.)}$$

For the liquid phase, the chemical potential can be expressed as following:

$$\begin{aligned} \mu_H^{(l)} &= \mu^0(T) + RT \ln a_w + [P_H - P_{sat}^0(T)]\bar{v}_w + \rho_w g H \bar{v}_w \\ &= \mu^0(T) - \Pi_H \bar{v}_w + [P_H - P_{sat}^0(T)]\bar{v}_w + \rho_w g H \bar{v}_w \end{aligned} \quad \text{(S3.)}$$

where ρ_w and $\bar{v}_w$ are the density and the molar volume of liquid water. The activity is linked to the osmotic pressure (Π_H) originated from the solute presence in the liquid (at $z = H$), i.e., $\Pi_H(C_{X,H}) = -\frac{RT}{\bar{v}_w} \ln a_w$.

As shown in Eq. (1) in the main text, the evaporative water vapor flux from the leaf pores, $J_w^{(v)}$, is expressed as follows:

$$J_w^{(v)} = k_{leaf}\left(P_H^{(v)} - P_\infty^{(v)}\right) \quad \text{(S4.)}$$

where $P_\infty^{(v)}$ is the partial pressures of vapor in the ambient outside the boundary layer of water vapor (at $z = z_\infty$), respectively; k_{leaf} is the mass transfer coefficient for the water vapor, and estimated using Eq. (10) in the main text.

The leaf-to-root membrane area ratio is defined as: $A_R \equiv A_{leaf} / A_{root}$ and the mass conservation leads to: $J_w = A_R J_w^{(v)}$, where J_w is the water flux based on the root membrane area. Requiring $\mu_H^{(v)} = \mu_H^{(l)}$ and combining with Eq. (S4), the hydraulic pressure in the leaf pore (P_H), which is negative in value is expressed as follows:

$$P_H = P_{sat}^0(T) + \frac{RT}{\bar{v}_w}\ln\frac{P_\infty^{(v)} + J_w/\left(A_R k_{leaf}\right)}{P_{sat}^0(T)} + \Pi_H - \rho_w g H \quad \text{(S5.)}$$

2) Stem (xylem conduit)

For a cylindrical tube with the diameter of xylem conduit (d_X), the momentum balance in z-direction shows:

$$0 = -\frac{\partial P}{\partial z} - \rho_w g + \eta\left[\frac{1}{r}\frac{\partial}{\partial r}\left(r\frac{\partial u_z}{\partial r}\right)\right] \quad \text{(S6.)}$$

where r is the variable in the radial coordinate, u_z is the fluid velocity in z-direction, and η is the dynamic viscosity of liquid water. The integration of Eq. (S6) from $r = 0$ to $r = d_X/2$ results in the average velocity:

$$\bar{U}_X = \frac{J_w}{\rho_w} = -\frac{d_X^2}{32\eta}\left(\frac{\partial P}{\partial z} + \rho_w g\right) \quad \text{(S7.)}$$

Integration of Eq. (S7) from $z = 0$ (xylem-root membrane interface) to $z = H$ (leaf pore) leads to the following expression for the hydraulic pressure at $z = 0$:

$$P_0 = P_H + \left(\frac{32\eta J_w}{\rho_w d_X^2} + \rho_w g\right)H \quad \text{(S8.)}$$

When the solute distribution reaches steady-state, the convective flux and diffusive flux need to be counterbalanced, which requires:

$$C_X(z) = C_{X,0}\exp\left(\frac{J_w}{\rho_w D_{eff}} z\right) \quad \text{(S9.)}$$

Assuming a perfect solute rejection by the root membrane and perfect solute retention by the meniscus in the leaf pore, the total mass of the solute in the xylem conduit is conserved, which requires:

$$\bar{C}_X H = \int_0^H C_X(z)\,dz = C_{X,0}\frac{\rho_w D_{eff}}{J_w}\left[\exp\left(\frac{J_w H}{\rho_w D_{eff}}\right) - 1\right] \quad \text{(S10.)}$$

where $\bar{C}_X$ is the volume-average solute concentration and D_{eff} is an effective diffusion coefficient that accounts for both molecular diffusion and Taylor dispersion: $D_{eff} = D_0(1 + Pe_d^2/196)$, with $Pe_d \equiv \frac{\bar{U}_X d_X}{D_0}$;[4] D_0 is the molecular diffusion coefficient of the solute. Accordingly, the solute concentrations at the xylem interface with the root membrane ($C_{X,0}$) and with the leaf pore ($C_{X,H}$) can be determined:

$$C_{X,0} = \bar{C}_X \frac{J_w H}{\rho_w D_{eff}}\left[exp\left(\frac{J_w H}{\rho_w D_{eff}}\right) - 1\right]^{-1} \quad \text{(S11.)}$$

$$C_{X,H} = C_{X,0}\, exp\left(\frac{J_w H}{\rho_w D_{eff}}\right) \quad \text{(S12.)}$$

3) Root membrane

From the Eq. (7) in the main text, the solution-diffusion mechanism can be written as:

$$J_w = A_m[(P_a - P_0) - (\Pi_{-\delta} - \Pi_0)] \quad \text{(S13.)}$$

where A_m is the water permeability of the membrane; the feed is exposed to the ambient pressure P_a; $\Pi_{-\delta}$ and Π_0 are the osmotic pressure at the feed-root membrane interface and the xylem-root membrane interface, respectively. Osmotic pressures are functions of solute concentrations, i.e., $\Pi_{-\delta} = \Pi_{-\delta}(C_{F,-\delta})$ and $\Pi_0 = \Pi_0(C_{X,0})$. The solute concentration at the feed-root membrane interface ($C_{F,-\delta}$) is elevated from the bulk concentration in the feed ($C_{F,-\infty}$) by concentration polarization[5]:

$$C_{F,-\delta} = C_{F,-\infty}\exp\left(\frac{J_w}{\rho_w k_{root}}\right) \quad \text{(S14.)}$$

The mass transfer coefficient in the feed (k_{root}) for turbulent flows is estimated as[43]:

$$Sh_{root} = 0.037 Sc_w^{1/3} Re_{L,-\infty}^{4/5} \quad \text{(S15.)}$$

where $Sh_{root} \equiv k_{root}L/D_0$, where L is the characteristic length over which the boundary layer of solute concentration develops at the feed-root membrane interface, which is equated to be the length scale of root membrane (e.g., length scale of building foundation), and L = 50 m is used as

a commonly observable length scale of urban buildings. Also, $Sc_w = \nu_w/D_0$ and $Re_{L,-\infty} = U_{-\infty}L/\nu_w$, where ν_w is the kinematic viscosity of water. Feed flow velocity ($U_{-\infty}$) of 1 m s^{-1} is assumed for calculation.

4) Solving by the iterative method

The Newton-Raphson iterative method was used to solve the coupled Eqs. (S1 – S15).[7] Specifically, in Eq. (13), P_0 is substituted by Eq. (S5) and (S8); $\Pi_{-\delta} = \Pi_{-\delta}(C_{F,-\delta})$ and $\Pi_0 = \Pi_0(C_{X,0})$ are substituted by Eq. (S11) and (S14). Through iterations, the water flux J_w is obtained, as well as $C_{X,0}$, $C_{X,H}$, P_0, P_H, and other parameters. Accordingly, The concentration polarization factor in the xylem conduit is determined (Fig. 4B). The saturation vapor pressure at the meniscus, $P_H^{(v)}$ is then determined, which is a function of the temperature, solute concentration ($C_{X,H}$), and hydraulic pressure (P_H), known as the Kohler equation [8, 9]:

$$P_H^{(v)}(T, C_{X,H}, P_H) = P_{sat}^0(T)\exp\left[\frac{(P_H - \Pi_H)\bar{v}_w}{RT}\right], \quad \text{(S16.)}$$

where $\Pi_H = \Pi_H(C_{X,H})$. Accordingly, the vapor pressure depression is determined (Fig. 4C). Knowing P_H, the contact angle between the meniscus and the leaf pore wall (θ) is also determined from the Young-Laplace equation (Eq. (1)), which is required to be larger than the receding contact angle (θ_{min} = 10° for hydrophilic surfaces considered here).

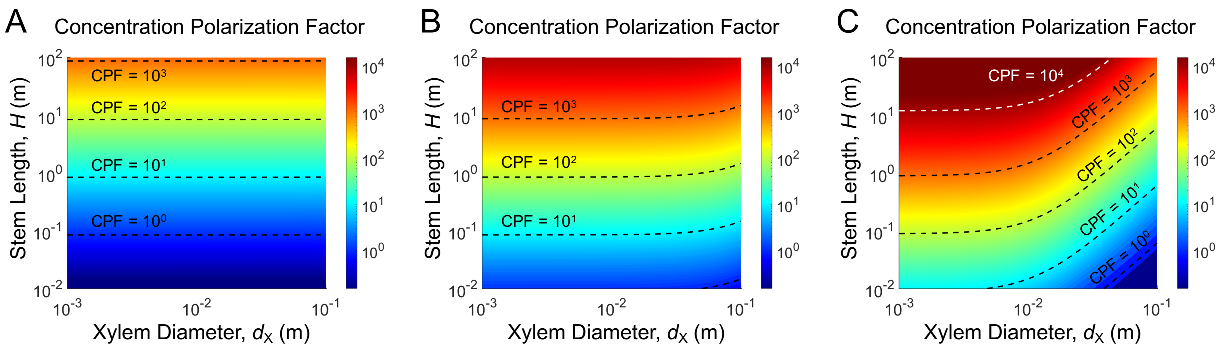

Fig. S1. Degree of concentration polarization in the xylem conduit determined by stem length (H) and xylem conduit diameter (d_X) for **(A)** $A_R = 1$; **(B)** $A_R = 10$; and **(C)** $A_R = 100$ (same as Fig. 3B). Concentration polarization factor (CPF) is defined as CPF ≡ $(C_{X,H} - C_{X,0})/\bar{C}_X$. $C_{F,-\infty} = 1$ M NaCl; $\bar{C}_X = 0.5$ mM is considered such that the maximum concentration of solutes in the xylem does not exceed 5 M.

Note S2. Heterogeneous nucleation for different negative pressures and xylem hydrophobicity

Surface hydrophobicity has a determining role for the propensity of cavitation, as a more hydrophobic surface reduces the free energy barrier to form bubble size larger than critical size. The probability of bubble formation from homogeneous nucleation is quite small.[10] Therefore, we only consider the heterogeneous nucleation here. The probability of the occurrence of a cavitation event following the formation of a vapor nucleus on a solid surface can be calculated using the classical nucleation theory. The nucleation rate, I, that is, the number of nucleation events per unit solid surface area per unit time is given as [10, 11]:

$$I = I_0 \exp\left[-\frac{\Delta G_{cav}}{kT}\right] \quad \text{(S17.)}$$

where I_0 is a kinetic prefactor and k is the Boltzmann constant. ΔG_{cav} is the free energy barrier for cavitation given as:

$$\Delta G_{cav} = \frac{16\pi\gamma^3}{3(\Delta P)^2}\frac{2 + 3\cos\theta_X - \cos^3\theta_X}{4} \quad \text{(S18.)}$$

Here, γ is the surface tension of liquid; θ_X is the contact angle between the liquid and the solid surface (e.g., inner surface of artificial xylem conduit); ΔP is the pressure difference between the liquid phase and the vapor phases (i.e., inside the bubble) across the liquid-vapor interface. The pressure in the vapor phase is essentially saturation vapor pressure determined by the temperature, curvature and solute concentration in the liquid phases[8, 9] (also in Eq. S16), of which magnitude is generally less than 1 bar. The pressure in the liquid phase can be negative for both natural and artificial trees, and large negative pressure will greatly reduce the free energy barrier for cavitation. Fig. S1 shows the free energy barrier for different magnitudes of ΔP and θ_X's, normalized by the free energy for $|\Delta P| = 1$ bar and $\theta_X = 0°$ ($\Delta G_{cav}/\Delta G_{cav,0}$). The two vertical lines at $\Delta P = 10$ bar and $\Delta P = 100$ bar indicate that the ΔP values are equivalent to the osmotic pressures of 0.2 M and 2 M NaCl, respectively. For instance, the negative pressure inside the artificial xylem conduit needs to be lowered at least below -100 bar in order to dewater 2 M NaCl feed. The water affinity of the artificial xylem conduit surface significantly influences the free energy barrier for cavitation. Compared to a perfectly hydrophilic surface ($\theta_X = 0°$), a highly hydrophobic surface ($\theta_X = 150°$)

has ~100 times lower free energy barrier, which greatly elevates the risk of cavitation. This again emphasizes that not only the xylem conduit surface needs to be hydrophilic, but also a very tight root membrane would be necessary to prevent any permeation of hydrophobic contaminants, onto which bubble formation may occur.

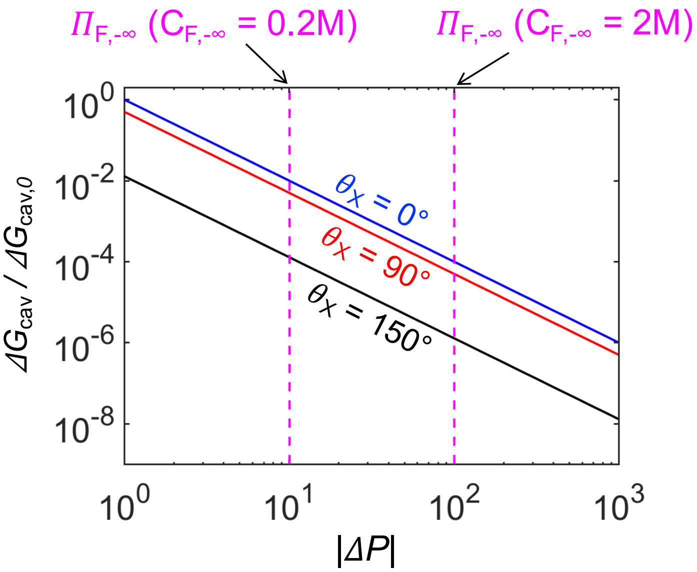

Fig. S2. Free energy barrier of cavitation for different magnitudes of pressure discontinuity across the liquid-vapor interface of bubble ($|\Delta P|$) and contact angles between the liquid and the xylem conduit inner surface (θ_X). The two vertical lines at $\Delta P = 10$ bar and $\Delta P = 100$ bar indicate that the ΔP values are equivalent to the osmotic pressures of 0.2 M and 2 M NaCl, respectively.

Note S3. Evolution of solute concentration profile in artificial xylem conduit

Due to low flow velocities in the artificial xylem conduits expected (<~O(10^{-5} m s^{-1}), Fig. 4 and 5), it takes a rather long time duration to reach the steady-state, solute concentration distribution. Neglecting radial distribution, the cross-section averaged, solute concentration distribution (C_X') in the xylem conduit along the z-direction can be determined based on the convection-diffusion equation:

$$\frac{\partial C_X'}{\partial t} = -\bar{U}_X \frac{\partial C_X'}{\partial z} + D_{eff} \frac{\partial^2 C_X'}{\partial z^2} \qquad \text{(S19.)}$$

When the time scale for convection is much larger than that for diffusion, i.e., $H/\bar{U}_X \gg H^2/D_{\text{eff}}$, the convection time scale ($H/\bar{U}_X$) may be taken to non-dimensionalize the time variable t. Taking $\tilde{t} \equiv t\bar{U}_X/H$, $\tilde{C}_X \equiv C_X'/\bar{C}_X$, $\tilde{z} \equiv z/H$, and $Pe_H = \frac{\bar{U}_X H}{D_{eff}}$, the Eq. (S19) can be non-dimensionalized as following:

$$\frac{\partial \tilde{C}_X}{\partial \tilde{t}} = -\frac{\partial \tilde{C}_X}{\partial \tilde{z}} + \frac{1}{Pe_H} \frac{\partial^2 \tilde{C}_X}{\partial \tilde{z}^2} \qquad \text{(S19.)}$$

We note from Eq. (S19) and Fig. 5 that when $Pe_{\text{H}} \gg 1$, the flow is dominated by convective flow. Eq. (S19) was numerically solved using the implicit Euler method for time integration and central difference for spatial discretization,[7] with the results for $Pe_{\text{H}} = 10$ shown in Fig. S2.

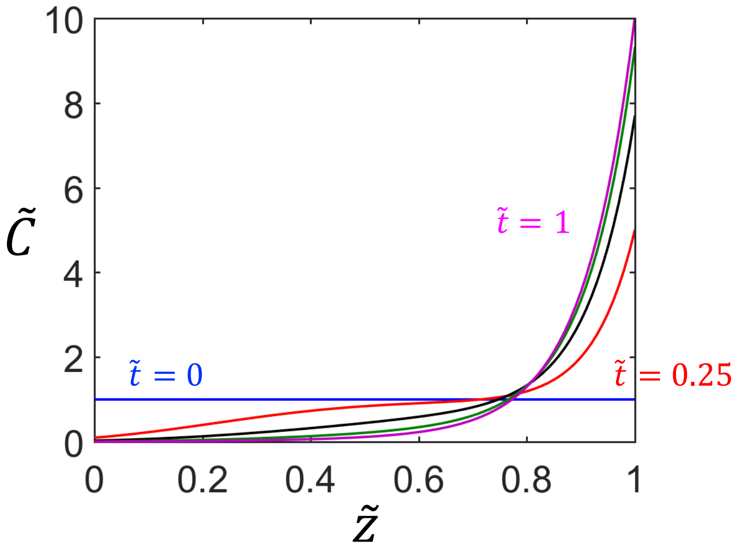

Fig. S3. Evolution of concentration distribution in the xylem conduit. At the initial stage ($\tilde{t} = 0$), the concentration is assumed to be uniform.

Driven by the convective flow, overwhelming the counter diffusive flow, the solute concentration at the leaf ($\tilde{z} = 1$) rapidly increases, and that at the root ($\tilde{z} = 0$) rapidly decreases. As an illustration, considering H =10 m and $\overline{U}_X$=10^{-5} m s^{-1}, the time scale of reaching a steady-state is estimated: $H/\overline{U}_X$~10^6 s ~O(100 days). Nevertheless, the fast decrease of the solute concentration at the xylem-root membrane interface indicates that the osmotic pressure is quickly negated, which eliminates the advantage of reducing the cavitation risk, and the analysis based on the steady-state concentrations (Eqs. (8) and (9)) provides conservative estimates on the water flux through the artificial tree.

Table S1. Nomenclature used in the main text

A_{leaf}	Leaf layer area (m^2)
A_{root}	Root membrane area (m^2)
A_R	Leaf-to-root membrane area ratio, $A_{\mathrm{R}} = A_{\mathrm{leaf}} / A_{\mathrm{root}}$
A_{m}	Water permeability of root membrane (kg m^{-2} h^{-1} bar^{-1} or L m^{-2} h^{-1} bar^{-1})
$C_{F,-\infty}$	Molar concentration of solute (NaCl) in the bulk of the feed (mol m^{-3} or mol L^{-1}) at z = $z_{-\infty}$
$C_{F,-\delta}$	Molar concentration of solute (NaCl) at the feed-root membrane interface (mol m^{-3} or mol L^{-1}) at z = -δ
$C_{X,0}$	Molar concentration of solute (NaCl) at the xylem-root membrane interface (mol m^{-3} or mol L^{-1}) at $z = 0$
$C_{X,H}$	Molar concentration of solute (NaCl) in the leaf pore (mol m^{-3} or mol L^{-1}) at $z = H$
$\bar{C}_X$	Volume averaged concentration in the xylem conduit (mol m^{-3} or mol L^{-1})
D_0	Molecular diffusion coefficient of solute (NaCl) (m^2 s^{-1})
D_{eff}	Effective diffusion coefficient of solute (NaCl) (m^2 s^{-1})
$D_w^{(v)}$	Molecular diffusion coefficient of water vapor (m^2 s^{-1})
d_{leaf}	Diameter of leaf pore (m)
d_{X}	Diameter of xylem conduit (m)
g	Gravitational acceleration (= 9.81 m s^{-2})
H	Stem length (m)

J_w	Mass flux of water through root membrane and xylem conduits (kg m^{-2} s^{-1})
$J_w^{(v)}$	Evaporative mass flux of water vapor (kg m^{-2} s^{-1})
k_{leaf}	Mass transfer coefficient for water vapor (s)
L	Length scale over which the boundary layer of water vapor develops (m)
M_w	Molar mass of water (= 1.8 × 10^{-2} kg mol^{-1})
P_a	Ambient pressure (10^5 Pa)
P_0	Hydraulic pressure at the xylem-root membrane interface (Pa)
P_H	Hydraulic pressure in the leaf pore (Pa)
P_{sat}^0	Saturation vapor pressure associated with a meniscus of pure water with zero curvature (Pa)
$P_H^{(v)}$	Saturation vapor pressure at the meniscus in the leaf pore (Pa)
$P_\infty^{(v)}$	Partial pressure of water vapor in the ambient outside of the water vapor concentration boundary layer (Pa)
R	Universal gas constant (= 8.31 J mol^{-1} K^{-1})
ρ_w	Density of liquid water (= 10^3 kg m^{-3})
T	Temperature
$\bar{U}_X$	Average velocity of water flow in the xylem conduit (m s^{-1})
U_∞	Air flow velocity (m s^{-1})
$U_{-\infty}$	Feed flow velocity (m s^{-1})
$\bar{v}_w$	Molar volume of liquid water (= 1.8 × 10^{-5} m^3 mol^{-1})
Greek letters	

γ	Surface tension of water (N m^{-1} or J m^{-2})
δ	Root membrane thickness (m)
ε	Leaf layer porosity
η	Dynamic viscosity of liquid water (Pa s)
θ	Contact angle between the meniscus and leaf pore wall (°)
θ_{min}	Receding contact angle between the meniscus and leaf pore wall (°)
ν_a	Kinematic viscosity of air (m^2 s^{-1})
$\mu_H^{(l)}$	Chemical potential of liquid-phase water at the meniscus in the leaf pore (J mol^{-1})
$\mu_H^{(v)}$	Chemical potential of vapor-phase water at the meniscus in the leaf pore (J mol^{-1})
μ^0	Chemical potential of pure water at standard condition (J mol^{-1})
Π_H	Osmotic pressure in the leaf pore (Pa)
$\Pi_{-\delta}$	Osmotic pressure at the feed-root membrane interface (Pa)
Π_0	Osmotic pressure at the xylem-root membrane interface (Pa)
$\Pi_{-\delta}$	Osmotic pressure at the feed-root membrane interface (Pa)
$\Pi_{-\infty}$	Osmotic pressure in the bulk feed (Pa)
Non-dimensional numbers	
Pe_d	Péclet number based on xylem diameter ($\equiv \frac{\bar{U}_X d_X}{D_0}$)
Pe_H	Péclet number based on stem length ($\equiv \frac{\bar{U}_X H}{D_{eff}}$)
Re_L	Reynolds number ($= U_\infty L/\nu_a$)
Sc	Schmidt number ($= \nu_a/D_w^{(v)}$)

Sh_{leaf}	Sherwood number
Abbreviations	
CPF	Concentration polarization factor
RH	Relative humidity
RO	Reverse osmosis

REFERENCES

1. Lee, J.; Laoui, T.; Karnik, R., Nanofluidic transport governed by the liquid/vapour interface. *Nat Nanotechnol* **2014,** *9*, (4), 317-323.
2. Hoch, G.; Chauhan, A.; Radke, C. J., Permeability and diffusivity for water transport through hydrogel membranes. *J Membrane Sci* **2003,** *214*, (2), 199-209.
3. Nobel, P. S., *Physicochemical and environmental plant physiology*. 3rd ed.; Elsevier Academic Press: Amsterdam ; Boston, 2005; p xx, 567 p.
4. Brenner, H.; Edwards, D. A., *Macrotransport processes*. Butterworth-Heinemann: Boston, 1993; p xxiv, 714 p.
5. Tiraferri, A.; Yip, N. Y.; Straub, A. P.; Romero-Vargas Castrillon, S.; Elimelech, M., A method for the simultaneous determination of transport and structural parameters of forward osmosis membranes. *J Membrane Sci* **2013,** *444*, 523-538.
6. Gekas, V.; Hallström, B., Mass transfer in the membrane concentration polarization layer under turbulent cross flow. I. Critical literature review and adaptation of existing sherwood correlations to membrane operations. *J Membrane Sci* **1987,** *30*, (2), 153-170.
7. Moin, P., *Fundamentals of Engineering Numerical Analysis*. 2 ed.; Cambridge University Press: Cambridge, 2010.
8. Wex, H.; Stratmann, F.; Topping, D.; McFiggans, G., The Kelvin versus the Raoult Term in the Kohler Equation. *J Atmos Sci* **2008,** *65*, (12), 4004-4016.
9. Lee, J.; Straub, A. P.; Elimelech, M., Vapor-gap membranes for highly selective osmotically driven desalination. *J Membrane Sci* **2018,** *555*, 407-417.
10. Holtta, T.; Vesala, T.; Peramaki, M.; Nikinmaa, E., Relationships between embolism, stem water tension, and diameter changes. *J Theor Biol* **2002,** *215*, (1), 23-38.
11. Kanduc, M.; Schneck, E.; Loche, P.; Jansen, S.; Schenk, H. J.; Netz, R. R., Cavitation in lipid bilayers poses strict negative pressure stability limit in biological liquids. *P Natl Acad Sci USA* **2020,** *117*, (20), 10733-10739.
